# Identifying hotspots and risk factors for tick-borne encephalitis virus emergence at its range margins to guide interventions, Great Britain

**DOI:** 10.2807/1560-7917.ES.2025.30.13.2400441

**Published:** 2025-04-03

**Authors:** Richard MJ Hassall, Maya Holding, Jolyon M Medlock, Festus A Asaaga, Sophie O Vanwambeke, Roger Hewson, Bethan V Purse

**Affiliations:** 1 UK Centre for Ecology and Hydrology, Benson Lane, Crowmarsh Gifford, Wallingford, United Kingdom; 2 Virology and Pathogenesis Group, Specialist Microbiology and Laboratories, UK Health Security Agency, Porton Down, Salisbury, United Kingdom; 3 National Institute for Health and Care Research (NIHR) Health Protection Research Unit in Emerging and Zoonotic Infections at the University of Liverpool, Liverpool, United Kingdom; 4 Medical Entomology and Zoonoses Ecology, Climate Change and Health Security, UK Health Security Agency, Porton Down, Salisbury, United Kingdom; 5 Université catholique de Louvain (UCLouvain), Earth & Life Institute, Earth and Climate Research Center, Louvain-la-Neuve, Belgium; 6 Faculty of Infectious and Tropical Diseases, London School of Hygiene & Tropical Medicine, London, United Kingdom

**Keywords:** Tick-borne encephalitis, surveillance, risk maps

## Abstract

**Background:**

Tick-borne encephalitis virus (TBEV) is expanding its range in Europe, with increasing human cases reported. Since the first detection of TBEV in ticks in the United Kingdom in 2019, one possible, two probable and two confirmed autochthonous cases in humans have been reported.

**Aim:**

We aimed to understand the environmental and ecological factors limiting TBEV foci at their range edge and predict suitable areas for TBEV establishment across Great Britain (GB) by modelling patterns of exposure to TBEV in deer.

**Methods:**

We developed spatial risk models for TBEV by integrating data between 2018 and 2021 on antibodies against tick-borne flavivirus in fallow, muntjac, red and roe deer with data on potential risk factors, including climate, land use, forest connectivity and distributions of bank voles and yellow-necked mice. We overlayed modelled suitability for TBEV exposure across GB with estimations on number of visitors to predict areas of high human exposure risk.

**Results:**

Models for fallow, muntjac and roe deer performed well in independent validation (Boyce index > 0.92). Probable exposure to TBEV was more likely to occur in sites with a greater percentage cover of coniferous woodland, with multiple deer species, higher winter temperatures and rates of spring warming.

**Conclusion:**

The resulting TBEV suitability maps can be used by public health bodies in GB to tailor surveillance and identify probable high-risk areas for human exposure to guide awareness raising and vaccination policy. Combining animal surveillance and iterative spatial risk modelling can enhance preparedness in areas of tick-borne disease emergence.

**Key public health message**

**What did you want to address in this study and why?**

Tick-borne encephalitis virus (TBEV) was first detected in the United Kingdom (UK) in 2019. Deer have been exposed to the virus, and it has been found in ticks. Human cases have also been reported. We aimed to identify and map conditions favouring this virus by relating locations of past TBEV infection in deer to different habitats and climates in Great Britain (GB).

**What have we learnt from this study?**

We found that TBEV infections were more likely in areas of GB with more coniferous woodland, more deer species present, higher winter temperatures and faster temperature rises in spring. We identified suitable areas for TBEV across GB, which will help us better understand current risk and how these risks may change in the future. To better understand risk there is a need for improved tests to differentiate between TBEV and other closely related viruses.

**What are the implications of your findings for public health?**

We produced maps to identify the overlap of areas suitable for TBEV and for people to visit. This provided information on where people may be at higher risk of contracting TBEV. This is important to guide public health planning and awareness and to target further surveillance for TBEV in GB, within ticks, animals and humans. This work can inform national policy groups that assess the risk of new and emerging infections in GB and other countries.

## Introduction

Tick-borne encephalitis (TBE) is a severe acute neuro-infection of humans, caused by a tick-borne flavivirus (tick-borne encephalitis virus, TBEV), with over 50,000 human cases reported in Europe in the last decade [1].Tick-borne encephalitis is seasonally linked to host-seeking activity of *Ixodes* tick vector species (mostly nymphs) with humans usually becoming infected via a bite from an infected tick but also through consumption of infected raw milk products [2,3].

Tick-borne encephalitis virus is widely distributed across Europe and Asia, but its presence is highly focal, even within the distribution of its competent tick vectors *Ixodes ricinus* and *Ixodes persulcatus* in Europe and Asia. The complex ecology and focal distribution make TBEV very difficult to control. Patients with TBE mainly receive supportive treatment. Preventive measures include vaccination, community awareness raising and tick-bite prevention measures [4]. An improved understanding and mapping of the conditions favouring TBEV foci is essential for spatial targeting of these available TBE preventative measures, especially public awareness [5,6]. This is even more imperative given the increased incidence of TBE across Europe and emergence of new TBEV foci in previously unaffected countries [7-10]. In 2019, TBEV was detected in England [6,11,12] and since then, five possible, probable or confirmed human cases of TBEV that were acquired in the United Kingdom (UK) have been reported [13,14].

Natural cycles of TBEV in Europe occur within forest and adjoining meadow biotypes, and small mammals such as bank voles (*Myodes glareolus*) and yellow-necked mice (*Apodemus flavicollis*) have been implicated in the maintenance of transmission [15,16]. Deer species may also play an important role in the TBEV cycle as a key reproduction host for *I. ricinus*, contributing to the maintenance of tick populations. Moreover, multiple transmission mechanisms are involved, including systemic transmission between viraemic small mammal hosts and ticks, and co-feeding transmission between nymphs and larvae feeding in close proximity on the same hosts [17]. An additional mechanism, trans-ovarial transmission, is often disregarded, with its contribution to the maintenance of TBEV being unclear [17,18]. Humans are accidental dead-end hosts.

The factors driving the highly focal distribution of TBEV are not well understood, especially in new areas of emergence. The presence of the tick vector, reservoir and reproduction hosts are not sufficient for the maintenance of TBEV. Wide-ranging climate, landscape and host factors have been linked to patterns in transmission and emergence in different parts of Europe [19]. These include variation in temperature, humidity, precipitation as well changes in land cover such as forest and wildlife host densities, in addition to human behaviours in and around forests underpinning exposure. Most studies of patterns in TBEV have leveraged human case data and as such capture human exposure, which is likely to represent only the tip of the iceberg in terms of the conditions in which transmission occurs. Integrating data on exposure of wildlife sentinels and ticks into surveillance systems, alongside human surveillance, may provide a more complete picture of the factors limiting TBEV distribution and risk areas. Since 2018, the UK Health Security Agency (UKHSA) has conducted large-scale monitoring of tick-borne flaviviruses in four deer species and ticks from varying habitats [11,20] to capture a wide range of conditions that favour transmission. This scheme indicated that exposure of deer to TBEV is highly focal and limited to few areas in Great Britain (GB). Genomic sequence analysis revealed existence of two European TBEV strains, that are mostly closely related to strains from Norway and the Netherlands [6,11,12].

Despite effort in modelling TBEV risk [19], as is commonly noted across zoonotic diseases, the feedthrough of these models and maps into disease control policy has been rather limited [21,22]. To address this disconnect for TBEV in the GB context, our partnership involves key actors and networks in surveillance and preparedness for tick-borne diseases, to help frame and tailor model outputs for decision making as part of a participatory co-production process [23,24] of the TickSolve project (https://ticksolve.ceh.ac.uk/).

In this study, we combined UKHSA national surveillance data on patterns in exposure of four deer species to tick-borne flaviviruses, with abiotic and biotic environmental data, to model and map the conditions restricting current TBEV foci in GB. We aimed to (i) quantify the role of climate, landscape and host factors in restricting TBEV foci in GB compared with other areas of TBEV circulation in Europe, (ii) develop maps of TBEV hazard across GB and integrate patterns in human recreational use to understand where human exposure risk may be highest with key actors in public health responses, (iii) validate the potential impact of these risk maps, as well as linkage of tick and wildlife surveillance with modelling, on decision-making for TBEV mitigation.

## Methods

### Epidemiological and environmental data

We used geolocated results from ELISA testing of 3,348 blood samples from 1,307 sites from fallow deer (*Dama dama*), muntjac deer (*Muntjac reevesi*), red deer (*Cervus elaphus*) and roe deer (*Capreolus capreolus*) in England and Scotland [11]. These samples were collected from wild culled deer by deer managers, who were culling as part of routine management practice. They also recorded associated information about the deer species and location, between February 2018 and March 2021. The serum samples were tested for antibodies to TBEV using Immunozym FSME IgG All Species ELISA (PROGEN Biotechnik GmbH, Heidelberg, Germany), following manufacturer’s instructions and cut-offs. Positive test results indicate exposure to the TBEV-serocomplex. Due to close genetic homology between TBEV and louping ill virus (LIV), previous testing was carried out on 1,309 samples with titres ≥ 20 using a LIV hemagglutination inhibition test for discriminatory testing [11]. Cohen’s κ indicated substantial agreement (0.61) between the methods, therefore, it was not possible to use these methods to discriminate between TBEV and LIV [6,11,12]. Given this diagnostic limitation, we refer throughout to probable TBEV exposure to reflect the uncertainty in serological specificity. 

The data were summarised, per deer species, as presence or absence of ELISA-positive deer at a 1 × 1 km resolution across England and Scotland and used as the response variable in models. To identify environmental drivers of exposure to TBEV in deer, environmental predictors previously related to tick dynamics, tick and TBEV hazard and host distribution were selected a priori and included humidity and temperature [25], terrain roughness [26], land cover [27], woodland connectivity within a 1 km and 2 km buffer around each 1 km grid cell, as well as presence of suitable habitat for potential TBEV hosts, bank voles, yellow-necked mice and deer [20,28]. The latter layers were the probability of multiple small mammals or multiple deer being present, calculated by summing across probability of presence outputs for each individual species from [20,28]. Further information about these predictors such as sources, biological rational for inclusion and processing can be found in Supplementary Material S1.

### Predicting hotspots of deer exposure to tick-borne encephalitis virus and associated environmental factors

To predict the probability of TBEV presence or establishment, we used a boosted regression tree (BRT) approach. Boosted regression trees have performed well when compared with other methods used to fit species distribution models, which is likely due to their ability to fit complex nonlinear relationships [29]. On the assumption that TBEV has recently emerged in GB, it may not have filled all the suitable habitats or niches for transmission, as is often assumed for the application of species distribution models (SDM). However, given that TBEV transmission is occurring across various parts of GB [11], describing the current realised niche of TBEV using an SDM will still provide a conservative estimate of the current conditions favouring transmission to inform risk assessments.

Given that deer species differ in their behaviour, habitat associations and range across GB, we fitted separate sets of BRTs to understand drivers of TBEV exposure for each deer species. The models were fitted with tree complexity of 4 and learning rate adjusted to ensure the number of trees exceeds 1,000 for all deer species [30]. We fitted 100 models for each deer species keeping a 1:1 ratio of absences and presences as recommended [31]. For each model, we resampled absence data to obtain the same number of absences as positives. The sampling of absences was weighted by the number of samples taken from the focal site, leading to preferential selection of absence sites with greater number of samples available.

We performed a comparison of different BRTs to deal with collinearity between variables and to select the most appropriate buffer size for woodland connectivity. To select a buffer for woodland connectivity in the model, we ran two sets of models for each deer species, one set of models with a 1 km buffer and another set with a 2 km buffer and selected the model with the greatest percentage of deviance explained. Furthermore, we did not fit any models where two explanatory variables had a Pearson’s correlation coefficient > 0.7 to avoid issues with multicollinearity [32]. Mean woodland patch area within a cell and percentage of coniferous woodland cover were highly correlated for all deer species (r = 0.68–0.89). We opted to use percentage of coniferous woodland cover in all models alongside percentage of deciduous woodland cover to allow us to determine the influence of different woodland types on exposure to TBEV. For the sites in the roe deer and red deer datasets, there was also a high correlation between small mammal occupancy and spring warming rate. We thus ran four sets of models for these deer species - two models to select buffer size excluding small mammal occupancy and two models to select buffer size excluding spring warming rate. For all models, we checked for spatial autocorrelation in the residuals using Moran’s I correlograms. We then selected a final model based on % deviance explained and the mean area under the curve (AUC) from using a 10-fold cross-validation approach [30]. Independent validation of prediction maps was also carried out with 124 additional positive samples using the Boyce index (fallow = 71, muntjac = 22, roe = 31) that were independent from the data used in the models. The Boyce index evaluates how well habitat suitability values from the model predict evaluation data using a precited-to-expected ratio (P/E ratio). This index ranges from -1 to 1 and a positive estimate, with a monotonic increase in P/E ratio in relation to increasing habitat suitability, indicates that model predictions are consistent with the distribution of presences in the evaluation dataset [33].

The ELISA also detected exposure to LIV, has resulted in seropositivity in deer being detected in areas with known LIV, and therefore probable seroconversions to LIV in these circumstances. We addressed this by down-weighting the contribution of those presence sites falling into areas where LIV has been recorded, using site level weights. We obtained data on 556 LIV diagnoses recorded in cattle and sheep at the postcode level from the UK Animal and Plant Health Agency (APHA) across 145 postcode districts from 2001 to 2023. To maintain confidentiality in this dataset, cases from postcode districts that contained < 5 holdings were assigned to the closest postcode district with > 5 holdings. This meant that some postcode districts may have had LIV cases but were listed as having no cases. Overall, there were 47 instances where this occurred and a total of 11 postcode districts had cases assigned to them from unknown nearby postcode districts. None of the positive deer samples fell into these 11 postcode districts. To account for anonymisation of the data and for potential variation in reporting effort across postcodes, we interpolated across postcodes by assigning each postcode with no cases the mean number of reported cases of LIV in all neighbouring postcode districts. This spatial interpolation was used to calculate the site weights in the model. For details on calculation of number of reported LIV cases and site weights please see Supplementary Material S2.

We used model predicted probability of presence outputs to produce GB scale predictions of the relative likelihood of TBEV occurrence or establishment (as estimated from probable TBEV exposure in deer). As each deer species has a different distribution across GB and cannot be exposed to TBEV where they are absent, we restricted geographical predictions of exposure to TBEV based on predicted probability of occurrence for each deer species from Croft et al. [20]. Any site with a relative probability of presence of < 0.1 for a focal deer species was masked. This limits predictions of TBEV suitability to the approximate range of different deer species by excluding areas with extremely low probability of occurrence but we note that some predictions may be in areas outside of the existing range of focal deer species. To generate a prediction of TBEV suitability incorporating modelled exposure for all deer species, we calculated the mean probability of TBEV exposure at each site across predictions for all deer species.

### Identifying areas of potential human exposure

To highlight areas where human recreation overlaps with areas where TBEV is likely to be present or establish, we used model outputs from Hooftman et al. [34] that predict recreational demand across GB. This modelling framework incorporates travel distance and the predicted frequency with which people are likely to visit a site to estimate the recreational demand in each site. Sites that people visit once a week captures estimated demand in sites that people use for local general recreation whereas sites that people visit once a year captures areas that people visit less frequently and travel further to reach. We used these two visit frequencies as they represent different types of recreation and different at-risk groups within GB that could be targeted with human surveillance and risk communication efforts. We used bivariate maps to plot the relationship between recreation demand and likelihood of TBEV occurrence or establishment.

To outline the areas with highly suitable sites in GB, we summed the number of highly suitable sites and estimated number of visits within Counties and Unitary Authorities across GB [35].

## Results

Blood samples were received from 361 sites for fallow deer (52 positive), 228 sites for muntjac deer (38 positive), 822 sites for roe deer (56 positive) and 352 sites for red deer (38 positive). Positives were detected in both England and Scotland and most positives (66%) were located in Norfolk, Hampshire and Suffolk.

Across all model sets, performance of models was relatively similar, and all models selected performed well in cross-validation (AUC > 0.8) and independent validation (Boyce index > 0.92), apart from the red deer model which had a relatively poor predictive performance (AUC = 0.64), presented in Supplementary Table S3.1. As such, we present results for predicted TBEV exposure of muntjac, fallow and roe deer and interpret these in relation to patterns in human recreation and provide results for red deer in Supplementary Figures S4.1, S4.2 and S4.3.

**Figure 1:**
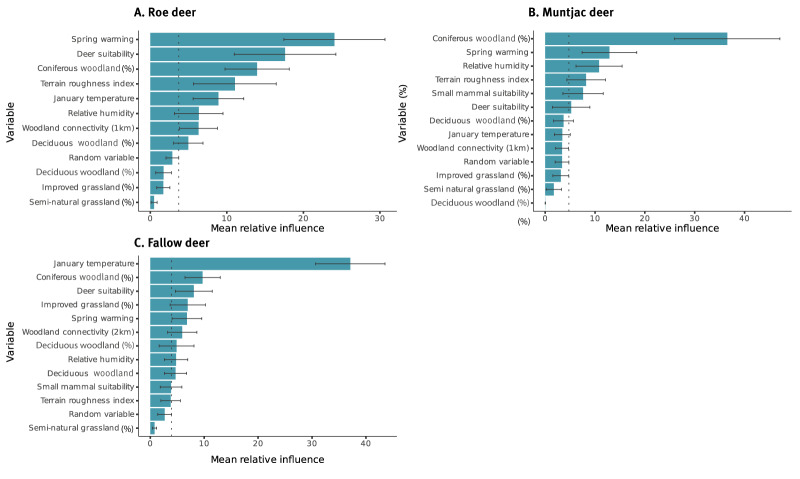
Mean relative influence of environmental variables included in the model on tick-borne encephalitis virus with standard deviation across model runs, Great Britain, 2018–2021

**Figure 2:**
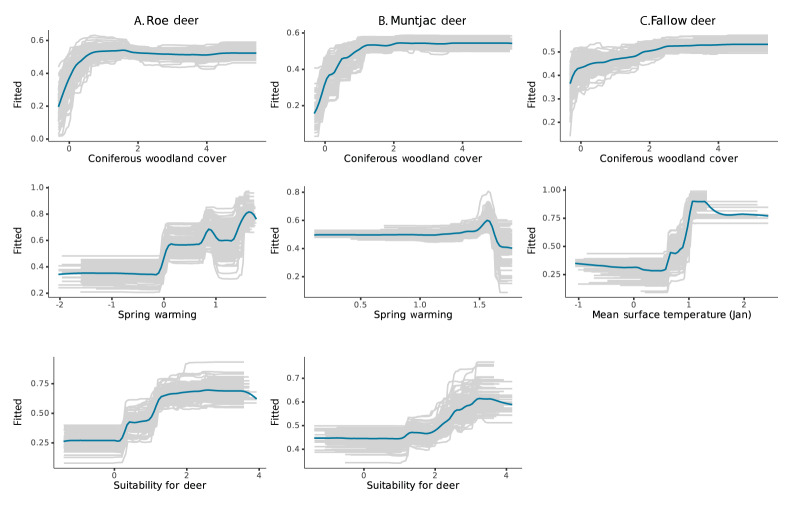
Modelled relationship between probability of exposure of fallow deer, muntjac deer and roe deer to tick-borne encephalitis virus and coniferous woodland cover, temperature-related variables and deer occupancy, Great Britain, 2018–2021

Using BRTs means the relative influence of predictors on the response, probable TBEV exposure, can be quantified. Temperature-related variables such as mean annual surface temperature and rate of spring warming (February to April in GB) had a high mean relative influence on the likelihood that deer are exposed to TBEV compared to other predictors (Figure 1). Percentage area of coniferous woodland was also an important factor influencing TBEV exposure, relative to other factors (Figure 1). Deer occupancy was also influential in the models fitted for roe and fallow deer (Figure 1A,1C). Overall, deer were more likely to have ELISA-positive test results with probable TBEV exposure in sites with high percentage cover of coniferous woodland, higher rate of spring warming or mean annual surface temperature and, in the case of fallow and roe deer, areas with higher probability of multiple deer species being present (Figure 2).

Dotted line shows upper standard deviation of relative influence values for a randomly generated variable. Variables to the right of the dotted line had greater influence than a randomly generated variable.

**Figure 3:**
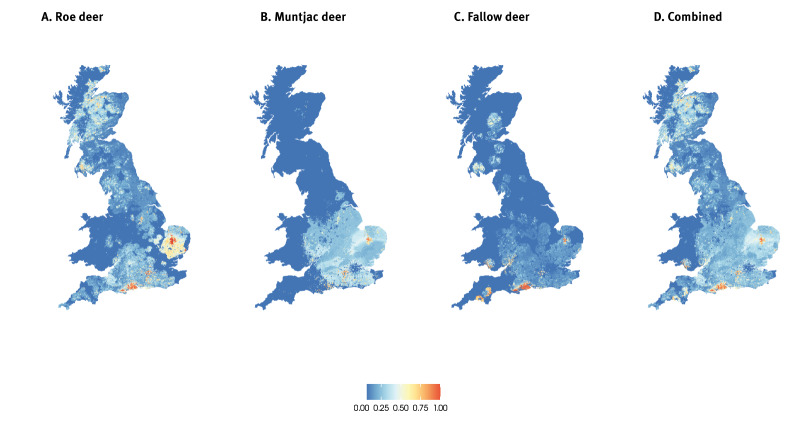
Predicted probability of occurrence or establishment of tick-borne encephalitis virus, based on probable exposure to TBEV in fallow, muntjac and roe deer, Great Britain

**Figure 4:**
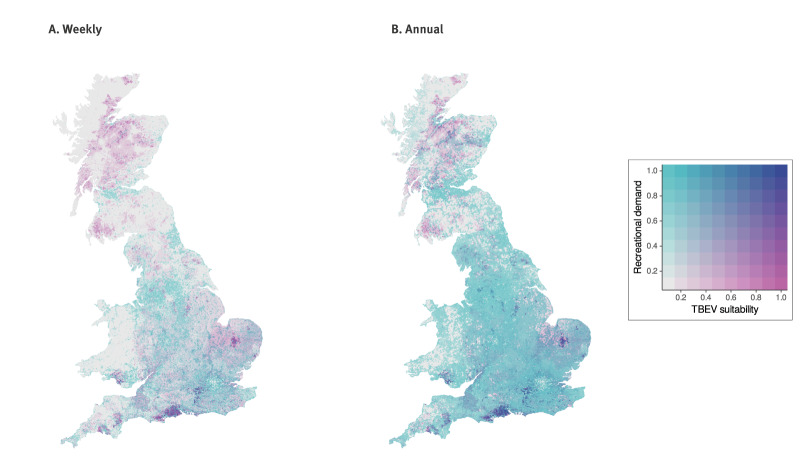
Bivariate maps showing overlap of predicted weekly and annual recreational demand (normalised between 0 and 1) and areas suitable for presence or emergence of tick-borne encephalitis virus, Great Britain

Using these models, we predicted the probability of TBEV occurrence or establishment based on deer with probable exposure to TBEV within GB and overlaid this with recreational demand in sites across GB based on estimations of weekly and yearly visits to identify areas where TBEV exposure risk and recreational demand is high across GB. Building on the current known distribution of deer exposure, our models identified additional highly suitable sites (> 0.75) in the south and east of England and south Wales (Table 1 and Figure 3). We also identified areas within these regions that have relatively high recreational demand and high suitability (Table 1 and Figure 4).

**Table 1:** Areas with suitable sites for transmission of tick-borne encephalitis virus to humans, Great Britain

Area	Highly suitable sites^a^	Predicted number of visits	
		Weekly	Annually^b^
Hampshire	297	6,797	6,445,666
Dorset	178	2,166	973,402
Norfolk	92	593	569,006
Suffolk	60	409	469,091
Highland	60	7	8,417
Neath Port Talbot	46	836	243,728
Bridgend	35	740	225,906
Surrey	30	1,444	937,206
Bournemouth, Christchurch and Poole^c^	24	504	396,306
Bracknell Forest	18	574	664,484
Swansea	18	463	122,179
Devon	17	234	77,455
Moray	13	1	476
Wiltshire	11	239	112,443

^a^ A highly suitable site was defined as sites with a suitability of > 0.75. Areas with > 10 highly suitable sites were included.

^b^ The weekly and annual numbers represent mutually exclusive visit types and therefore will not correspond to each other in this way. The weekly type of visit measures frequency per week of people visiting their local areas for recreation. The annual type of visit measures the number of people that visit an area once a year e.g. for holidays.

^c^ Bournemouth, Christchurch and Poole is considered a separate unitary authority.

TBEV: tick-borne encephalitis virus.

The legend shows relative suitability, probability of presence or establishment, for TBEV across Great Britain. Dark blue areas are predicted to have the lowest suitability and dark red areas the highest suitability.

TBEV: tick-borne encephalitis virus.

Light blue areas have higher recreational demand and lower TBEV suitability. Pink areas have higher TBEV suitability and lower recreational demand. Dark blue areas have both high recreational demand and high TBEV suitability.

Tests for spatial autocorrelation did show lower level but significant correlation in model residuals at distances up to 10 km (Moran’s I ≤ 0.34), as presented in Supplementary Table S3.3. We do not expect this level of spatial autocorrelation to impact the inferred relative role of environmental predictors in driving TBEV patterns. Since the Boyce index values from our independent validation are high (> 0.92), indicative of high out-of-fit predictive performance of the model, we also conclude that the low-level spatial autocorrelation is having minimal impact.

## Discussion

Given that TBEV is expanding into new foci in north-western Europe [6,11,12] and causing human infections at its range edge in Europe, it is critical to identify the environmental and ecological conditions that promote transmission and human exposure, to guide surveillance and interventions. For a highly focal pathogen like TBEV in a new area of emergence, modelling human cases may underestimate the suitable conditions and geographical area at risk of transmission. Human cases of TBE are likely a limited representation of the enzootic circulation of the virus, where wildlife cycles can go undetected [36]. This was demonstrated in Thetford Forest, GB, where no human cases have so far been reported, despite Holding et al. [11] finding 47% of deer had been exposed. Additionally, in areas where the virus is considered absent, TBE may not be identified as a differential diagnosis so cases may be missed. We therefore combined serological surveillance of exposure in four ecologically diverse wild deer species, with spatial risk models integrating wide-ranging climate, host and land use factors, to identify conditions favouring TBEV foci in GB and possible hotspots of risk to people.

Climate is known to strongly affect *I. ricinus* population dynamics and activity, and TBE incidence, with variable effects in different parts of Europe [19]. Our finding that higher spring warming rates, faster increase in temperature from February to April, underpin current GB TBEV foci is consistent with studies from other European countries, like Norway [37], Sweden [38,39], Switzerland [40], Latvia, Estonia and Lithuania [7]. A higher rate of spring warming is thought to stimulate larvae and nymphs to quest for a host simultaneously in spring and increase the rate of co-feeding transmission from nymphs to larvae, in addition to increasing numbers encountering viraemic rodents, amplifying TBEV [7]. Though some European studies have identified TBEV foci in areas with low January temperatures (−4.4°C–4°C) [7,37], long exposure to cold temperatures can be detrimental to survival of overwintering *I. ricinus,* particularly, where there is limited snow cover, which may provide insulation and increased humidity [41,42]. Suitability for TBEV in GB increased in areas with higher mean temperatures in January (4.8–8.1°C), which could contribute to TBEV persistence by increasing overwintering survival of nymphs.

Human TBE incidence and tick hazard have been linked to the presence and densities of both TBEV transmission hosts, particularly forest rodent species, yellow-necked mouse and bank vole, and ungulate reproductive hosts that support and amplify tick populations [43,44]. Our models indicated that in areas predicted to be highly suitable for multiple deer species in GB, a larger proportion of roe and fallow deer were exposed to TBEV, suggesting there could be stronger amplification of *I. ricinus* tick populations in areas with diverse, overlapping deer populations. However, the modelled occurrence of yellow-necked mice and bank voles had a minor influence on whether deer were exposed to TBEV, possibly due to the uncertainties associated with small mammal distribution models and perhaps the widespread distribution of bank voles in GB. Finer scale empirical data on the heterogeneity of densities and habitat use of these small mammals is probably required to detect an association between TBEV foci and small mammal populations [45].

Forests may provide suitable habitat for reproductive hosts like deer, small mammal transmission hosts and tick populations, and therefore increase their encounter rate, amplifying TBEV transmission [19]. We found that increased cover of coniferous forest was associated with increased likelihood of probable deer exposure to TBEV in GB, aligning with two studies in Germany linking coniferous forest cover and human TBE cases [46,47] but in contrast to studies in Sweden and Latvia linking broad-leaved or mixed forest with TBEV with cases in humans [48,49]. The ecological mechanisms underpinning the association between TBEV foci and coniferous forest cover in GB are not yet fully understood and would be best examined empirically with finer scale field studies of links between ungulate and small mammal community composition and forest structure and knock on impacts on tick abundance and TBEV prevalence in ticks and hosts [50]. Understanding habitat use by different deer species at finer scales would also aid in understanding whether exposure is likely to occur in coniferous woodlands. Key transmission hosts such as yellow-necked mice require mature broadleaved woodlands to thrive but data from Thetford Forest has shown bank voles are able to sustain the same densities in mature coniferous woodlands as in the nearby broadleaved forests [51]. It may also be that the mammal and avian biodiversity (and hence tick hosts) in coniferous forests in GB is lower than in deciduous forests, and so larval and nymphal feeding is more concentrated towards these rodent species and deer.

Due to its recent detection in GB and therefore likely quite recent introduction, TBEV may not have yet filled all suitable habitat for transmission in GB. However, the low levels of spatial autocorrelation in model residuals and the high performance of models (Boyce indices exceeding 0.92) when validated with independent data from subsequent surveillance years, gives confidence that the risk maps can be used as a (conservative) baseline estimate of current hotspots for exposure within the UK.

Aligning with best practice [24,52], it is critical to identify with stakeholders how models can inform decision-making linked to interventions, for which key agencies and actors, and over which key temporal and geographical scales. Box outlines the anticipated value of the model outputs identified during our co-production process by GB decision-makers and actors working in public health. In addition, it is imperative to be transparent about model limitations and ensure that models represent a concrete benefit over information already used to guide decision-making (beneficence) [52]. These risk models are correlative and relatively static (TBEV may not be in equilibrium in GB) and show considerable uncertainty at grid square level. The risk maps should be interpreted as indicating short-term (1–5 years) population level risks of TBEV exposure over broader areas (≥ 5 km), not individual or site-level risks at finer scales (≥ 1 km). The maps predict areas that are potentially suitable for TBEV exposure, but TBEV is not necessarily present in areas that are predicted to be highly suitable. The range of roe, fallow and muntjac does not cover the whole of GB and as such our results may not identify any TBEV hotspots outside of these ranges. Nevertheless, following the precautionary principle, areas predicted to be highly suitable by these outputs can be used at national scale by public health bodies to tailor future deer, tick and human surveillance and provide a more detailed picture of the current extent of TBEV enzootic transmission, particularly given the wide distribution of the tick vector [53]. This future surveillance could have implications in informing at a national and local level, likely risk areas where there may be a need for increased awareness among the public and physicians, and feed into decision-making on vaccination guidance. Given that the GB niche of TBEV transmission is expected to evolve over the coming decades, the risk maps will be updated and validated iteratively with new surveillance data (every 1–2 years) and risk factors in discussion with stakeholders. Whilst our current approach aims to reduce the influence of ELISA-positive deer in LIV areas, it does not fully resolve the limitations posed by serological cross-reactivity. There remains an urgent need to develop and validate diagnostic tools, preferably incorporating molecular confirmation, that can accurately discriminate TBEV from LIV and other flaviviruses within the TBEV-serocomplex. This is particularly important in regions like GB where multiple flaviviruses co-circulate and diagnostic ambiguity may compromise surveillance accuracy.

Box **Anticipated value of risk models of tick-borne encephalitis virus to guide interventions by key actors and agencies at different spatial or temporal scales, Great Britain**

**Model functionality:**

• Combined deer exposure risk maps (Figure 3),

• Overlay of hotspots of TBEV exposure with areas of high recreation (Figure 4).

**Epidemiological interpretation and temporal scale in the next 1-5 years:**

• Relative risk of wildlife and human exposure to TBEV,

• Population level relative risk of exposure to TBEV through recreation.

**Interventions that could be informed:**

• *National scale:*

o Identify hotspots of risk outside the current known distribution to be targeted for deer and tick surveillance,

o Inform vaccination policy.

o *Key agencies:*

▪ Public health bodies,

▪ Government advisory groups,

▪ Deer Management groups/Forestry Commission.

• *Local scale:*

o Inform localities (> 5–10 km^2^), in which people work or recreate within natural habitats at higher relative risk of human TBEV exposure to guide awareness raising (public, occupational groups, local general practitioners).

o *Key agencies:*

▪ Local health authorities,

▪ National Park authorities and national landscapes,

▪ Land managers,

▪ Large environmental land and forest managers (e.g. National Trust, Woodland Trust, Wildlife Trusts, Forestry Commission, Scottish Forestry).

TBEV: tick-borne encephalitis virus.

Integrating data on TBEV occurrence in other compartments such as ticks and reproductive hosts into spatial risk models and including social drivers of exposure [54,55], will be beneficial in identifying which conditions favour human exposure among those that favour tick hazard. The mapping of the coincidence of recreational activities and TBEV exposure risk may be more valuable for local public health authorities and individuals by understanding from where people travel to reach different recreational sites, and how awareness and adaptive actions against ticks and ultimately tick encounter rates [56] vary among recreational groups. Long-term, multi-decadal assessment of the evolving risk of TBEV exposure as woodland is expanded (under national environmental policies) and climate changes, required for national adaptation planning, will require mechanistic modelling approaches and empirical surveys that explicitly capture links between tick, host and pathogen demography and interactions and land use and environmental drivers [57]. As part of our co-production process, over the next 1–2 transmission seasons, the value of correlative risk mapping vs dynamic transmission models for tick-borne disease mitigation will be interrogated further with cross-sectoral stakeholders [24].

Surveillance, monitoring and prevention of TBE in areas of suspected TBEV emergence requires an integrated One Health approach. We suggest the following key components to this approach: (i) detection of possible unidentified foci of TBEV circulation through sentinel deer, or potentially small mammals [58], targeted in predicted high-risk areas which subsequently updates spatial risk modelling and mapping to identify other potential areas of circulation; (ii) utilisation of molecular and genomic confirmation in the tick vector or rodent reservoir in suspected foci, identified through sentinel surveillance and mapping of probable TBE cases; (iii) targeted human surveillance in locations of confirmed circulation including measures raising awareness among clinicians and public tick awareness campaigns; (iv) reviewing and, where necessary, updating clinical criteria for testing of patients with a history of tick bite.

## Conclusion

This study highlights the value of combining wildlife surveillance data and spatial risk modelling for mapping hotspots of TBEV in new areas of emergence to guide interventions. Our approach provides insights into the climate, land use and host drivers of TBEV occurrence on its north-western range margin and highlights areas in which high predicted risks of TBEV exposure coincide with high rates of recreational habitat use. To foster the uptake of tick-borne disease models into policy and as a model for future tick-borne disease modelling efforts, we carefully delineate the key actors and interventions that could be informed by these risk maps at national and local scales.

## Supplementary Data

620000 Supplementary Material
