# Supplementary material for: Identifying hotspots and risk factors for tick-borne encephalitis virus emergence at its range margins to guide interventions, Great Britain
Source: Euro Surveill. 2025 Apr 3;30(13):2400441. doi: 10.2807/1560-7917.ES.2025.30.13.2400441 (PMC11969960; doi:10.2807/1560-7917.ES.2025.30.13.2400441)
Supplement: Supplementary Material [file 24-00441_HASSALL_Supplement.pdf]

Supplementary Information

This supplementary material is hosted by Eurosurveillance *as supporting information alongside the article "**Identifying hotspots and risk factors for tick-borne encephalitis virus emergence at its range margins to guide interventions"**, on behalf of the authors, who remain responsible for the accuracy and appropriateness of the content. The same standards for ethics, copyright, attributions and permissions as for the article apply. Supplements are not edited by* Eurosurveillance *and the journal is not responsible for the maintenance of any links or email addresses provided therein.*

S1 Description of environmental and ecological data included in risk models

These included the rate of spring warming based on temperature data from 2005 to 2015. To calculate the rate of spring warming we fitted a generalisable additive model to daily mean temperature at a 1km resolution across the UK from February to April. The mean rate of change was calculated using the smoother for each year. The mean rate of change for all years was then calculated for each 1km grid cell in the UK (Supplementary material S1).

To include the probability of occurrence for relevant hosts for ticks and TBEV, the predicted probability of occurrence of roe, red, fallow and muntjac deer was determined using outputs from Croft et al. (Croft et al., 2019). These layers were stacked and probabilities of presence summed across deer species per grid cell to provide information on occupancy patterns for these four deer species across the UK. To include small mammal hosts that have previously been implicated as potential reservoirs for TBEV, we used predicted occupancy for bank voles and yellow-necked mice from Croft et al. (Croft et al., 2017) and summed these to provide information on the occupancy patterns for both of these potential host species.

We included land cover types obtained from the UKCEH 2020 land cover map to understand the influence of both woodland coverage and connectivity of woodland in focal sites to woodland in surrounding sites. To understand whether establishment of TBEV may be linked to connectivity to other woodland patches, we calculated connectivity metrics for woodland across the UK using a distance-weighted area based metric outlined by Moilanen & Nieminen, (2002) Eq1.

$$S_i = A_i \sum_{j \neq i} A_j \, exp(-d_{i,j}) \qquad \text{Eq1.}$$

Where the connectivity of the focal patch to other woodland patches (S_i) is determined using the area of a focal patch (A_i) or patches within a 1km grid cell and the area of all other patches (A_j) as well as the distance between the focal patch i and all other patches ($d_{i,j}$). This connectivity metric outlines how well-connected woodland within a focal cell is to other woodland patches within a specified radius. This meant that woodland within the focal cells that was not connected to any other patches within a radius or cells that did not contain any woodland had a connectivity score of zero. We calculated connectivity at two different scales to capture potential effects of woodland connectivity on deer reproductive hosts with larger home range sizes as well as effects on ticks, small mammals, and deer with smaller home ranges i.e. within a 1km radius including all adjacent cells and a 2km radius incorporating two adjacent cells surrounding the focal cell. To account for the area of woodland within the focal cell we calculated two measures, the total area of woodland patch area within a cell and the percentage of a cell covered by deciduous or coniferous woodland.

As well as woodland, we also calculated the percentage cover per cell of three other land cover types that are less favorable for ticks in the UK but in which ticks may still occur, namely semi-natural grassland, improved grassland and heathland.

S2: Weighting scheme to account for lack of specificity in flavivirus serology and Louping Ill Virus circulation

To determine weights for models for each deer species we used the number of LIV diagnoses reported in cattle and sheep as proxy for areas in the UK where LIV is likely to be circulating. We then calculated the number of reported LIV diagnoses in relation to livestock density in each grid cell using

$$R_i = \frac{c_i}{n_i} \qquad \text{Eq2.}$$

Where reports in relation to livestock density in postcode i (R_i) is determined using the number of diagnoses reported in a postcode district from 2001 to 2023 (c_i), the total cattle and sheep population in 2016 (n_i).

To determine livestock population sizes we combined cattle and sheep density across GB by combining agcensus+ data giving livestock density at a 5kmx5km resolution for England, Scotland and Wales in 2016 and summing the density of cattle and sheep in each postcode district. Where a postcode did not fully cover a grid square, we calculated the percentage cover of the grid square and reduced density based on the proportion of a grid square covered by a postcode district. To account for anonymisation of the data and to account for potential variation in reporting effort across postcodes we assigned each polygon with a value of 0 the mean value of all neighbouring postcode districts within 100m of the spatial extent of the focal postcode district. The weight for each site where deer had a positive test result was then assigned using Eq3. to obtain a value between 1 and 101.

$$W_i = \left| \frac{R_i}{max(R)} \times 100 - 101 \right| \text{ Eq 3.}$$

Where, W_i is the weight at site i. A weight of 1 is the lowest weight based on the maximum incidence across all postcodes where deer tested positive and a weight of 101 is the highest weight based on the lowest incidence across all sites where deer tested positive. All sites where deer tested negative were assigned a weight of 101.

S3: Additional model results and validation

Table S3.1: *% deviance explained and cross-validation AUC from different sets of models of TBEV exposure in deer species in the UK . Values in bold indicate models chosen to make geographical predictions of TBEV exposure for each species.*

	Fallow		Muntjac		Red		Roe	
Model set	% deviance	AUC	% deviance	AUC	% deviance	AUC	% deviance	AUC
Presence of small mammals + connectivity with 1000m buffer	-	-	-	-	**0.4669**	**0.64**	0.7691	0.88
Presence of small mammals + connectivity with 2000m buffer	-	-	-	-	0.4699	0.62	0.7657	0.88
Rate of spring warming + connectivity with 1000m buffer	-	-	-	-	0.4644	0.63	**0.8134**	**0.9**
Rate of spring warming + connectivity with 2000m buffer	-	-	-	-	0.4644	0.63	0.8155	0.89
Connectivity with 1000m buffer	0.7711	0.91	**0.5212**	**0.8**	-	-	-	-
Connectivity with 2000m buffer	**0.7797**	**0.91**	0.5185	0.8	-	-	-	-

Figure S3.2: Results from Boyce Index using final prediction maps for fallow, muntjac, roe and the combination of these three deer species.

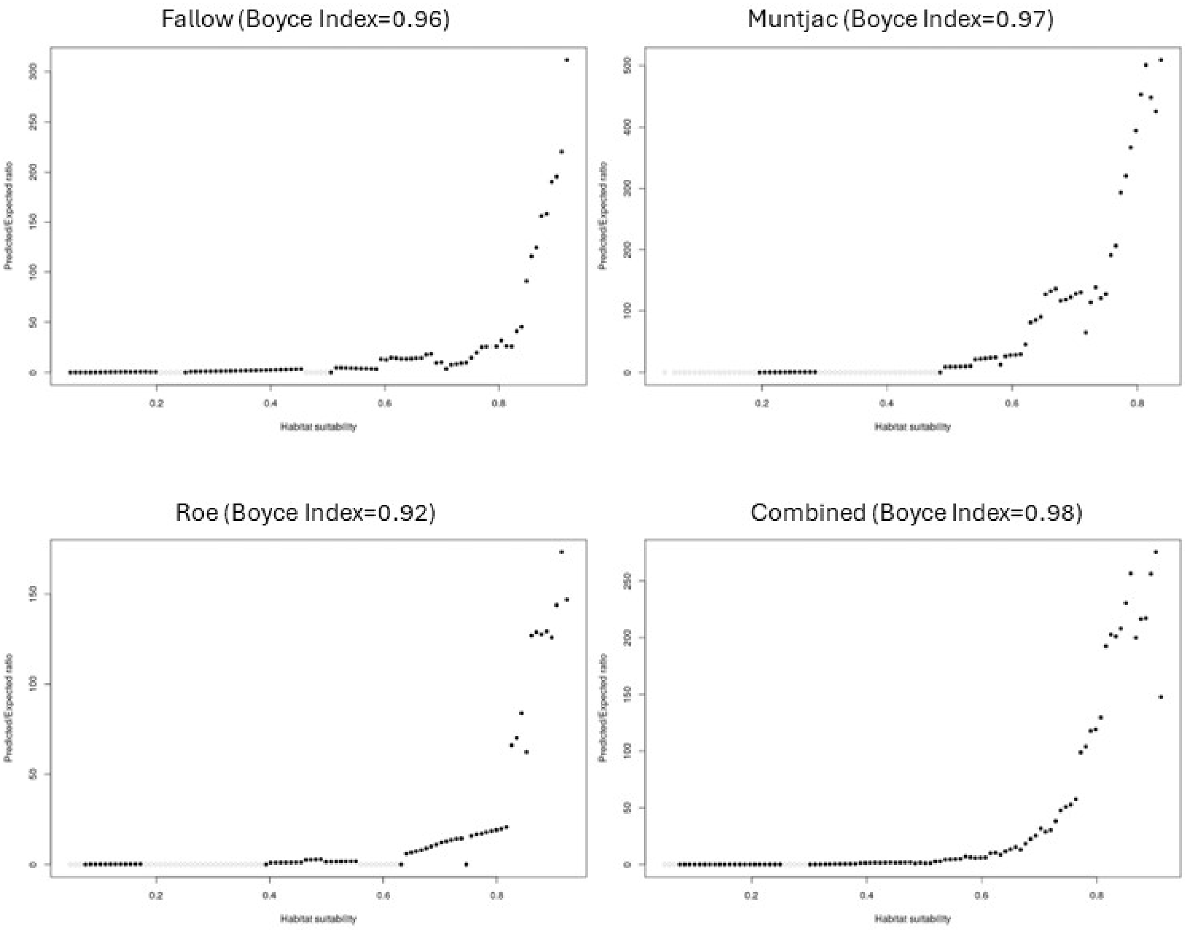

Table S3.3: Moran’s I on residuals from BRT models by distance bands. Table shows mean distance between points in a band, the mean and standard deviation of Moran’s I across model runs and the proportion of runs in with significant Moran’s I.

Species	Band (km)	Mean distance in band (km)	Mean Moran's i	SD Moran's i	Prop Significant
**Roe**	**0-10**	**5.47**	**0.21**	**0.07**	**0.95**
Roe	10-20	14.80	0.09	0.06	0.4
Roe	20-30	25.17	0.12	0.10	0.46
Roe	30-40	35.41	-0.04	0.12	0.13
Roe	40-50	46.99	-0.11	0.11	0.24
**Fallow**	**0-10**	**5.51**	**0.29**	**0.04**	**1**
Fallow	10-20	15.13	0.07	0.07	0.58
Fallow	20-30	25.07	-0.02	0.05	0.17
Fallow	30-40	34.81	-0.17	0.06	0.74
Fallow	40-50	45.77	-0.19	0.08	0.77
**Muntjac**	**0-10**	**6.05**	**0.34**	**0.09**	**1.00**
Muntjac	10-20	14.91	0.06	0.07	0.42
Muntjac	20-30	25.48	-0.38	0.09	0.99
Muntjac	30-40	35.71	-0.27	0.13	0.83
Muntjac	40-50	45.23	-0.28	0.15	0.70

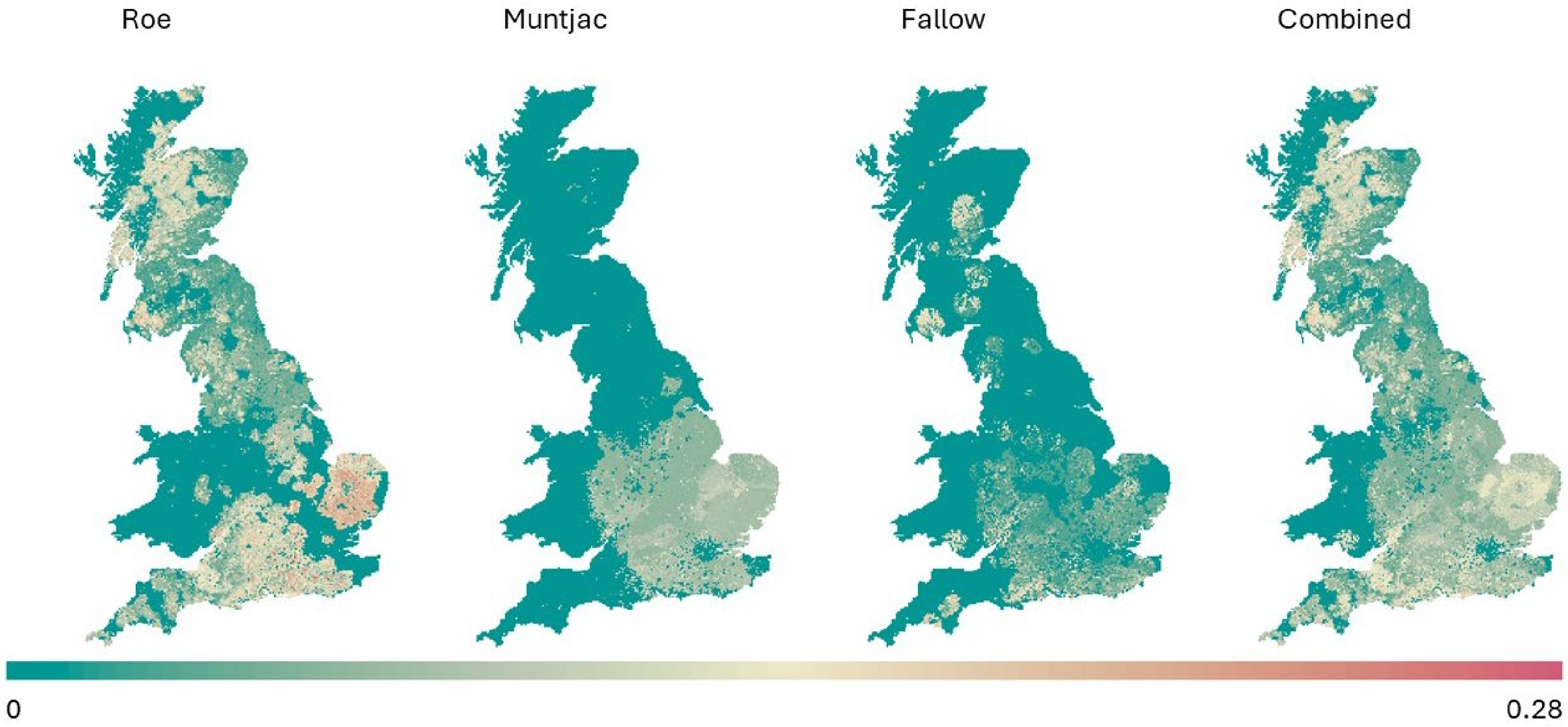

Figure S3.4: Standard deviation of suitability estimates across model runs for roe, muntjac, fallow and all species combined.

S4: Red deer results and combined maps of all deer species

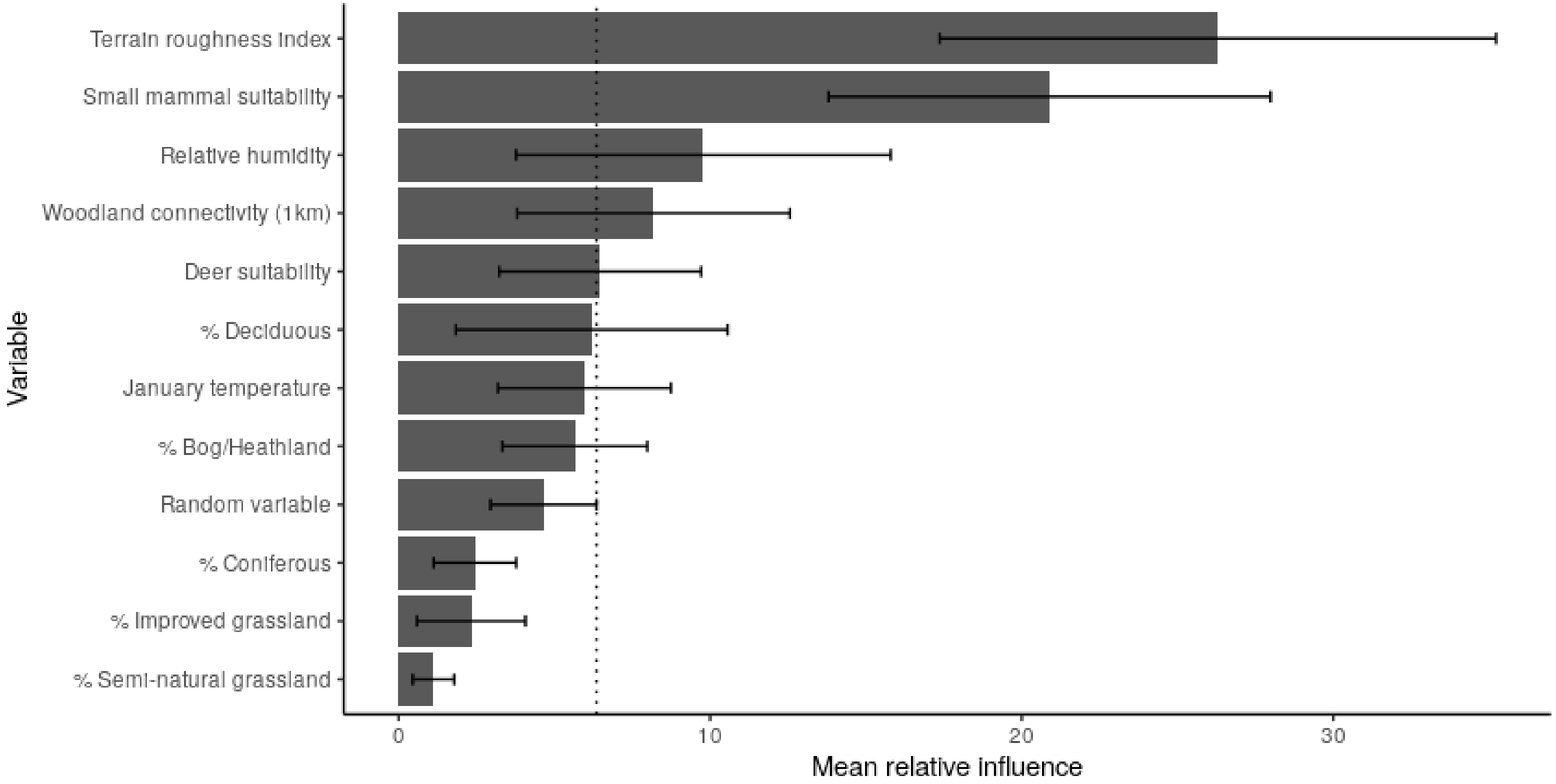

Figure S4.1: Mean relative influence of variables included in the model with standard deviation. Dotted line shows upper standard deviation of relative influence values for a randomly generated variable. Variables to the right of the dotted line had greater influence than a randomly generated variable.

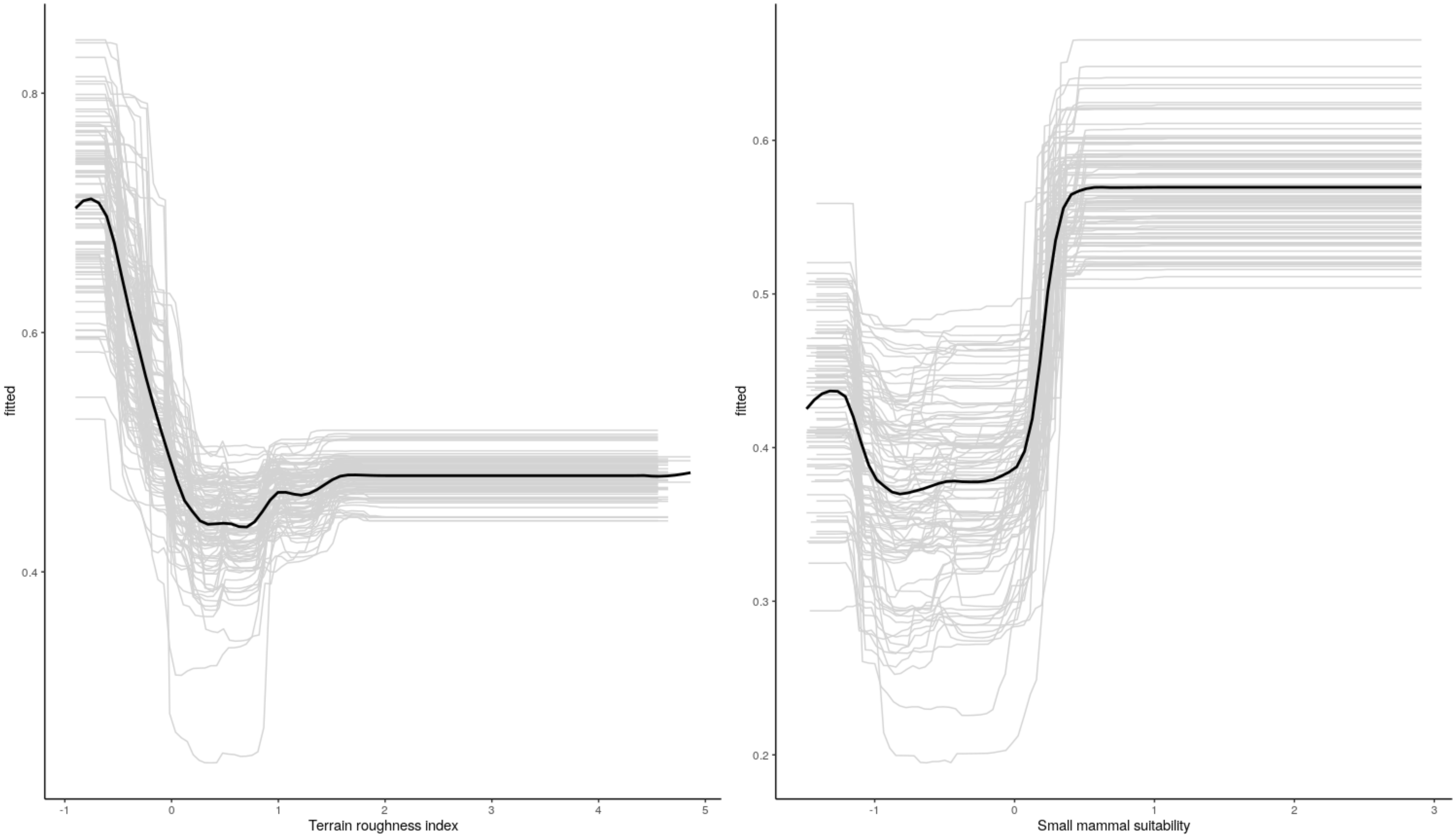

Figure S4.2: Relationship between probability of red deer being exposed to TBEV and Terrain roughness and small mammal suitability.

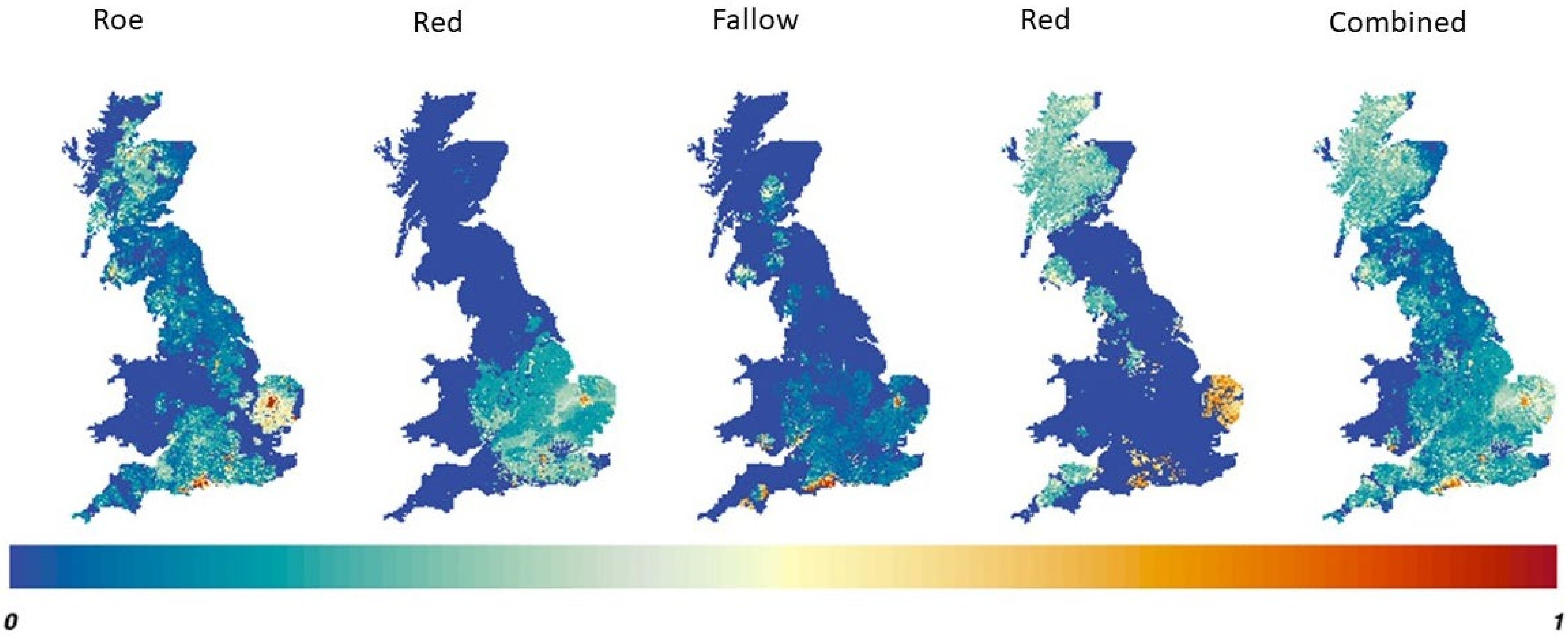

Figure S4.3: Predicted probability of TBEV occurrence or establishment based on exposure of fallow, muntjac, roe and red deer across GB.

Table S5: Counties with at least one highly suitable site. Table shows number of highly suitable sites (suitability >= 0.75) with total predicted number of visits based on weekly and annual use.

County	Highly suitable sites	Total predicted visits (weekly)	Total predicted visits (annual)
Hampshire	297	6797	6445666
Dorset	178	2166	973402
Norfolk	92	593	569006
Suffolk	60	409	469091
Highland	60	7	8417
Neath Port Talbot	46	836	243728
Bridgend	35	740	225906
Surrey	30	1444	937206
Bournemouth, Christchurch and Poole	24	504	396306
Bracknell Forest	18	574	664484
Swansea	18	463	122179
Devon	17	234	77455
Moray	13	1	476
Wiltshire	11	239	112443
Cornwall	7	10	4138
West Sussex	6	63	108216
East Sussex	5	61	58630
Dumfries and Galloway	5	0	0
Vale of Glamorgan	3	79	17605

Rhondda Cynon Taf	3	116	33363
West Berkshire	2	70	18112
Wokingham	2	122	41694
South Ayrshire	2	0	0
Stirling	2	0	1804
Argyll and Bute	2	0	87
Perth and Kinross	2	0	0
North Lanarkshire	2	0	1804
Blackburn with Darwen	1	14	29990
Windsor and Maidenhead	1	15	39181
Southampton	1	100	37377
Gloucestershire	1	1	2640
Aberdeenshire	1	0	43
Powys	1	12	4315

References

Croft, S., M Chauvenet, A. L., & Smith, G. C. (2017). *A systematic approach to estimate the distribution and total abundance of British mammals*. https://doi.org/10.1371/journal.pone.0176339

Croft, S., Ward, A. I., Aegerter, J. N., & Smith, G. C. (2019). Modeling current and potential distributions of mammal species using presence-only data: A case study on British deer. *Ecology and Evolution*, *9*(15), 8724–8735. https://doi.org/10.1002/ECE3.5424

Moilanen, A., & Nieminen, M. (2002). SIMPLE CONNECTIVITY MEASURES IN SPATIAL ECOLOGY. *Ecology*, *83*(4), 1131–1145. https://doi.org/10.1890/0012-9658(2002)083[1131:SCMISE]2.0.CO;2

Morton, R. D., Marston, C. G., O'Neil, A. W., & Rowland, C. S. (2021). Land Cover Map 2020 (25m rasterised land parcels, GB). . *NERC EDS Environmental Information Data Centre.* https://doi.org/https://doi.org/10.5285/6c22cf6e-b224-414e-aa85-900325baedbd

Riley, S. J., DeGloria, S. D., & Elliot, R. (1999). A terrain ruggedness index that quantifies topographic heterogeneity. *Intermountain Journal of Sciences*, *5*(1–4), 23–27.

Robinson, E. L., Blyth, E. M., Clark, D. B., Comyn-Platt, E., & Rudd, A. C. (2020). Climate hydrology and ecology research support system meteorology dataset for Great Britain (1961-2017) [CHESS-met]. . *NERC Environmental Information Data Centre.* https://doi.org/https://doi.org/10.5285/2ab15bf0-ad08-415c-ba64-831168be7293
